# Bis{2-[(2-furylmeth­yl)imino­meth­yl]-5-meth­oxy­phenolato-κ^2^ *N*,*O*}zinc(II)

**DOI:** 10.1107/S1600536811011536

**Published:** 2011-03-31

**Authors:** Chunyan Li

**Affiliations:** a College of Health Science, Wuhan Institute of Physical Education, Wuhan 430079, People’s Republic of China

## Abstract

In the title complex, [Zn(C_13_H_12_NO_3_)_2_], the Zn^II^ ion is located on a twofold rotation axis and is coordinated by two bidentate Schiff base ligands in a distorted tetra­hedral environment. The complex mol­ecules are stacked in columns along the *b* axis through C—H⋯O hydrogen bonds.

## Related literature

For the biological activity and applications of Zn(II) complexes, see: Csaszar *et al.* (1985 ▶); Greener *et al.* (1996 ▶); Gultneh *et al.* (1996 ▶); Aoki & Kimura (2004 ▶). For applications of furfuryl­amine derivatives, see: Camejo *et al.* (1992 ▶); Ledovskikh & Camejo (1993 ▶). For a related structure, see; Cai *et al.* (2010 ▶). 
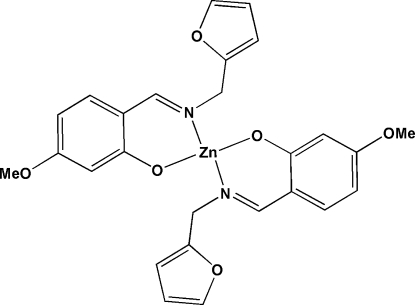


## Experimental

### 

#### Crystal data

[Zn(C_13_H_12_NO_3_)_2_]
*M* *_r_* = 525.84
Monoclinic, 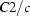

*a* = 27.210 (4) Å
*b* = 5.2244 (7) Å
*c* = 19.007 (3) Å
β = 119.507 (2)°
*V* = 2351.5 (5) Å^3^
*Z* = 4
Mo *K*α radiation
μ = 1.09 mm^−1^
*T* = 298 K
0.32 × 0.20 × 0.13 mm

#### Data collection

Bruker SMART CCD area detector diffractometer
Absorption correction: multi-scan (*SADABS*; Bruker, 2000 ▶) *T*_min_ = 0.722, *T*_max_ = 0.871
6855 measured reflections
2303 independent reflections
2062 reflections with *I* > 2σ(*I*)
*R*_int_ = 0.026

#### Refinement

*R*[*F* ^2^ > 2σ(*F* ^2^)] = 0.049
*wR*(*F* ^2^) = 0.128
*S* = 1.01
2303 reflections
160 parameters
1 restraint
H-atom parameters constrained
Δρ_max_ = 0.64 e Å^−3^
Δρ_min_ = −0.31 e Å^−3^

### 

Data collection: *SMART* (Bruker, 2000 ▶); cell refinement: *SAINT* (Bruker, 2000 ▶); data reduction: *SAINT*; program(s) used to solve structure: *SHELXTL* (Sheldrick, 2008 ▶); program(s) used to refine structure: *SHELXTL*; molecular graphics: *SHELXTL*; software used to prepare material for publication: *SHELXTL*.

## Supplementary Material

Crystal structure: contains datablocks global, I. DOI: 10.1107/S1600536811011536/is2670sup1.cif

Structure factors: contains datablocks I. DOI: 10.1107/S1600536811011536/is2670Isup2.hkl

Additional supplementary materials:  crystallographic information; 3D view; checkCIF report

## Figures and Tables

**Table 1:** Hydrogen-bond geometry (Å, °)

*D*—H⋯*A*	*D*—H	H⋯*A*	*D*⋯*A*	*D*—H⋯*A*
C9—H9*A*⋯O1^i^	0.97	2.32	3.292 (6)	177

Symmetry code: (i) 
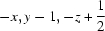
.

**Fig. 1.**
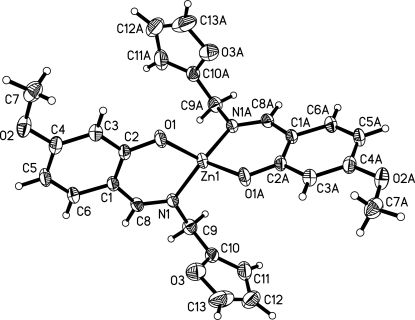
The molecular structure of the title compound, with the atom labeling scheme. Displacement ellipsoids are drawn at the 30% probability level. The suffix A corresponds to symmetry code - x, y, 1/2 - z.

**Fig. 2.**
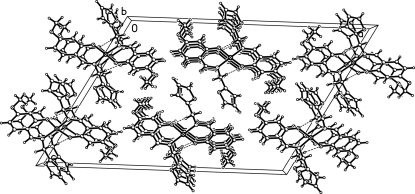
A packing diagram of the title compound, viewed along the b axis.

## supplementary crystallographic information

### Comment

Zinc(II) complexes with the chelate ligands are extensively investigated as models for the active site of carbonic anhydrase and other hydrolytically active enzymes (Greener *et al.*, 1996; Gultneh *et al.*, 1996). Zinc(II) complexes are also studied on the role of zinc(II) structural properties in protein folding (Aoki & Kimura, 2004). Interestingly, zinc(II) complexes are becoming important in pharmaceutical, dye, plastic industries and for liquid crystal technology (Csaszar *et al.*, 1985). In addition, the furfuryl amine and its derivatives are widely used as bioactive antibacterial agents and as steel corrosion inhibitors in recent research (Ledovskikh & Camejo, 1993; Camejo *et al.*, 1992). By taking the biological importance of furfurylamine into account, we designed the title complex containing nitrogen-oxygen donor atoms coordinated with zinc(II).

The title complex reported here is the mononuclear zinc(II) complex of Schiff-base ligand, derived from the condensation of 4-methoxysalicylaldehyde and furfuryl amine (Fig. 1). The zinc(II) atom has a distorted tetrahedral coordination formed by two N atoms and two O atoms from two Schiff-base ligands. The bond distances of Zn—O and Zn—N are 1.925 (3) and 1.984 (3) Å, respectively. The dihedral angle between the coordination planes (N1/Zn1/O1 and N1A/Zn1A/O1A) is 87.24 (8)° (symmetry code A: - *x*, *y*, 1/2 - *z*), which is slightly larger than that of 84.43 (6)° between the corresponding coordination planes around zinc(II) atom observed in the similar Schiff base zinc(II) complex, bis(3,5-dibromo-N-benzylsalicylaldiminato-N,O)zinc(II) (Cai *et al.*, 2010). The O1—Zn1—O1 angle is 109.27 (18)° and the N1—Zn1—N1 angle is 120.08 (18)°. The other angles subtended at the Zn(II) ion in (ZnN_2_O_2_)  is in the range of 96.69 (11)–117.49 (12)°.

In the crystal structure, the molecules are linked *via* intermolecular C—H···O hydrogen bonds forming a column along the *b* axis (Fig. 2).

### Experimental

4-Methoxysalicylaldehyde (304 mg, 2 mmol) and furfurylamine (194 mg, 2 mmol) were dissolved in an aqueous methanol solution (25 mL).The mixture was stirred at room temperature for 1 h to give a clear yellow solution, which was added to a solutionof Zn(NO_3_)_2_.6H_2_O (298 mg, 1 mmol) in methanol (10 mL). The mixture was stirred for 30 min at room temperature to give a yellow solution and then filtered. The yellow single crystals suitable for X-ray analysis were obtained by slowly evaporating the above filtrate at room temperature. The crystals were isolated and dried in a vacuum desiccator containing anhydrous CaCl_2_, in about 71% yield. Anal. Calcd for C_26_H_24_ZnN_2_O_6_: C 59.38, H 4.60, N 5.33%. Found: C 59.20, H 4.73 N, 5.30%.

### Refinement

All the H atoms were placed in geometrically idealized positions and constrained to ride on their parent atoms, with C—H distances of 0.93–0.97 Å, and with *U*_iso_(H) = 1.2*U*_eq_(C) or 1.5*U*_eq_(methyl C). A rigid bond restraint was applied for atoms C11 and C12.

### Crystal data

**Table:**

[Zn(C_13_H_12_NO_3_)_2_]	*F*(000) = 1088
*M**_r_* = 525.84	*D*_x_ = 1.485 Mg m^−^^3^
Monoclinic, *C*2/*c*	Mo *K*α radiation, λ = 0.71073 Å
Hall symbol: -C 2yc	Cell parameters from 2246 reflections
*a* = 27.210 (4) Å	θ = 2.5–23.7°
*b* = 5.2244 (7) Å	µ = 1.09 mm^−^^1^
*c* = 19.007 (3) Å	*T* = 298 K
β = 119.507 (2)°	Block, yellow
*V* = 2351.5 (5) Å^3^	0.32 × 0.20 × 0.13 mm
*Z* = 4	

### Data collection

**Table:**

Bruker SMART CCD area detector diffractometer	2303 independent reflections
Radiation source: fine-focus sealed tube	2062 reflections with *I* > 2σ(*I*)
graphite	*R*_int_ = 0.026
φ and ω scans	θ_max_ = 26.0°, θ_min_ = 2.2°
Absorption correction: multi-scan (*SADABS*; Bruker, 2000)	*h* = −33→33
*T*_min_ = 0.722, *T*_max_ = 0.871	*k* = −6→6
6855 measured reflections	*l* = −23→20

### Refinement

**Table:**

Refinement on *F*^2^	Primary atom site location: structure-invariant direct methods
Least-squares matrix: full	Secondary atom site location: difference Fourier map
*R*[*F*^2^ > 2σ(*F*^2^)] = 0.049	Hydrogen site location: inferred from neighbouring sites
*wR*(*F*^2^) = 0.128	H-atom parameters constrained
*S* = 1.01	*w* = 1/[σ^2^(*F*_o_^2^) + (0.0784*P*)^2^ + 2.4971*P*] where *P* = (*F*_o_^2^ + 2*F*_c_^2^)/3
2303 reflections	(Δ/σ)_max_ < 0.001
160 parameters	Δρ_max_ = 0.64 e Å^−^^3^
1 restraint	Δρ_min_ = −0.31 e Å^−^^3^

### Special details

**Table:**

Geometry. All esds (except the esd in the dihedral angle between two l.s. planes) are estimated using the full covariance matrix. The cell esds are taken into account individually in the estimation of esds in distances, angles and torsion angles; correlations between esds in cell parameters are only used when they are defined by crystal symmetry. An approximate (isotropic) treatment of cell esds is used for estimating esds involving l.s. planes.
Refinement. Refinement of F^2^ against ALL reflections. The weighted R-factor wR and goodness of fit S are based on F^2^, conventional R-factors R are based on F, with F set to zero for negative F^2^. The threshold expression of F^2^ > 2sigma(F^2^) is used only for calculating R-factors(gt) etc. and is not relevant to the choice of reflections for refinement. R-factors based on F^2^ are statistically about twice as large as those based on F, and R- factors based on ALL data will be even larger.

### Fractional atomic coordinates and isotropic or equivalent isotropic displacement parameters (Å^2^)

**Table:**

	*x*	*y*	*z*	*U*_iso_*/*U*_eq_	
Zn1	0.0000	0.78566 (12)	0.2500	0.0427 (3)	
N1	0.02638 (12)	0.5960 (6)	0.18435 (18)	0.0394 (7)	
O1	0.06578 (11)	0.9989 (6)	0.30670 (18)	0.0549 (7)	
O2	0.24426 (12)	1.3537 (7)	0.3745 (2)	0.0665 (9)	
O3	−0.03914 (17)	0.7542 (6)	0.0242 (2)	0.0729 (10)	
C1	0.11535 (15)	0.8329 (7)	0.2413 (2)	0.0403 (8)	
C2	0.10942 (14)	1.0010 (7)	0.2954 (2)	0.0402 (8)	
C3	0.15270 (16)	1.1795 (8)	0.3399 (3)	0.0465 (9)	
H3	0.1490	1.2918	0.3750	0.056*	
C4	0.20061 (16)	1.1895 (8)	0.3319 (3)	0.0489 (9)	
C5	0.20638 (16)	1.0283 (9)	0.2781 (3)	0.0550 (10)	
H5	0.2382	1.0385	0.2720	0.066*	
C6	0.16505 (16)	0.8560 (9)	0.2348 (3)	0.0505 (10)	
H6	0.1694	0.7483	0.1993	0.061*	
C7	0.2397 (2)	1.5304 (9)	0.4279 (3)	0.0675 (13)	
H7A	0.2080	1.6411	0.3976	0.101*	
H7B	0.2736	1.6309	0.4545	0.101*	
H7C	0.2345	1.4386	0.4675	0.101*	
C8	0.07515 (15)	0.6467 (7)	0.1911 (2)	0.0417 (8)	
H8	0.0851	0.5491	0.1591	0.050*	
C9	−0.00946 (17)	0.4112 (8)	0.1224 (2)	0.0508 (9)	
H9A	−0.0260	0.2943	0.1446	0.061*	
H9B	0.0134	0.3118	0.1061	0.061*	
C10	−0.05497 (17)	0.5418 (8)	0.0510 (2)	0.0517 (10)	
C11	−0.1095 (2)	0.4911 (15)	0.0025 (3)	0.0929 (17)	
H11	−0.1303	0.3563	0.0065	0.111*	
C12	−0.1294 (3)	0.6947 (16)	−0.0583 (4)	0.102 (2)	
H12	−0.1661	0.7157	−0.1007	0.123*	
C13	−0.0861 (3)	0.8419 (15)	−0.0423 (3)	0.103 (2)	
H13	−0.0874	0.9855	−0.0721	0.123*	

### Atomic displacement parameters (Å^2^)

**Table:**

	*U*^11^	*U*^22^	*U*^33^	*U*^12^	*U*^13^	*U*^23^
Zn1	0.0333 (4)	0.0510 (4)	0.0525 (4)	0.000	0.0278 (3)	0.000
N1	0.0346 (15)	0.0452 (16)	0.0426 (16)	0.0013 (13)	0.0222 (13)	0.0020 (13)
O1	0.0396 (15)	0.0674 (18)	0.0709 (18)	−0.0102 (13)	0.0372 (14)	−0.0200 (15)
O2	0.0414 (16)	0.070 (2)	0.083 (2)	−0.0157 (14)	0.0268 (16)	−0.0085 (18)
O3	0.079 (2)	0.070 (2)	0.064 (2)	0.0066 (17)	0.0316 (19)	0.0094 (16)
C1	0.0326 (18)	0.047 (2)	0.045 (2)	0.0031 (15)	0.0226 (16)	0.0047 (16)
C2	0.0301 (17)	0.0457 (19)	0.047 (2)	0.0029 (14)	0.0205 (15)	0.0023 (16)
C3	0.038 (2)	0.047 (2)	0.054 (2)	−0.0002 (16)	0.0232 (18)	−0.0007 (17)
C4	0.0342 (19)	0.052 (2)	0.056 (2)	−0.0027 (16)	0.0188 (18)	0.0068 (18)
C5	0.0336 (19)	0.074 (3)	0.066 (3)	0.0003 (18)	0.0304 (19)	0.005 (2)
C6	0.037 (2)	0.065 (3)	0.057 (2)	0.0034 (18)	0.0289 (18)	−0.002 (2)
C7	0.059 (3)	0.060 (3)	0.068 (3)	−0.018 (2)	0.019 (2)	−0.003 (2)
C8	0.0398 (19)	0.047 (2)	0.044 (2)	0.0051 (15)	0.0252 (17)	0.0000 (16)
C9	0.052 (2)	0.045 (2)	0.060 (2)	−0.0084 (18)	0.031 (2)	−0.0046 (19)
C10	0.043 (2)	0.065 (3)	0.050 (2)	−0.0058 (18)	0.0245 (18)	−0.014 (2)
C11	0.050 (3)	0.155 (5)	0.079 (3)	−0.021 (3)	0.036 (3)	−0.049 (3)
C12	0.064 (4)	0.167 (7)	0.056 (3)	0.042 (4)	0.015 (3)	−0.015 (3)
C13	0.119 (6)	0.113 (5)	0.055 (3)	0.052 (5)	0.027 (4)	0.014 (3)

### Geometric parameters (Å, °)

**Table:**

Zn1—O1^i^	1.925 (3)	C4—C5	1.391 (6)
Zn1—O1	1.925 (3)	C5—C6	1.357 (6)
Zn1—N1	1.984 (3)	C5—H5	0.9300
Zn1—N1^i^	1.984 (3)	C6—H6	0.9300
N1—C8	1.296 (4)	C7—H7A	0.9600
N1—C9	1.463 (5)	C7—H7B	0.9600
O1—C2	1.306 (4)	C7—H7C	0.9600
O2—C4	1.362 (5)	C8—H8	0.9300
O2—C7	1.422 (6)	C9—C10	1.479 (6)
O3—C13	1.360 (7)	C9—H9A	0.9700
O3—C10	1.375 (5)	C9—H9B	0.9700
C1—C2	1.422 (5)	C10—C11	1.332 (6)
C1—C6	1.422 (5)	C11—C12	1.465 (10)
C1—C8	1.423 (5)	C11—H11	0.9300
C2—C3	1.411 (5)	C12—C13	1.311 (11)
C3—C4	1.386 (6)	C12—H12	0.9300
C3—H3	0.9300	C13—H13	0.9300
			
O1^i^—Zn1—O1	109.27 (18)	C1—C6—H6	118.6
O1^i^—Zn1—N1	117.49 (12)	O2—C7—H7A	109.5
O1—Zn1—N1	96.69 (11)	O2—C7—H7B	109.5
O1^i^—Zn1—N1^i^	96.69 (11)	H7A—C7—H7B	109.5
O1—Zn1—N1^i^	117.49 (12)	O2—C7—H7C	109.5
N1—Zn1—N1^i^	120.08 (18)	H7A—C7—H7C	109.5
C8—N1—C9	117.3 (3)	H7B—C7—H7C	109.5
C8—N1—Zn1	120.4 (3)	N1—C8—C1	128.1 (3)
C9—N1—Zn1	122.1 (2)	N1—C8—H8	115.9
C2—O1—Zn1	125.6 (2)	C1—C8—H8	115.9
C4—O2—C7	118.4 (3)	N1—C9—C10	111.1 (3)
C13—O3—C10	107.1 (5)	N1—C9—H9A	109.4
C2—C1—C6	117.5 (3)	C10—C9—H9A	109.4
C2—C1—C8	125.7 (3)	N1—C9—H9B	109.4
C6—C1—C8	116.7 (3)	C10—C9—H9B	109.4
O1—C2—C3	117.7 (3)	H9A—C9—H9B	108.0
O1—C2—C1	123.4 (3)	C11—C10—O3	110.5 (5)
C3—C2—C1	118.9 (3)	C11—C10—C9	133.4 (5)
C4—C3—C2	120.8 (4)	O3—C10—C9	116.0 (4)
C4—C3—H3	119.6	C10—C11—C12	104.7 (6)
C2—C3—H3	119.6	C10—C11—H11	127.6
O2—C4—C3	123.4 (4)	C12—C11—H11	127.6
O2—C4—C5	115.9 (4)	C13—C12—C11	107.5 (5)
C3—C4—C5	120.7 (4)	C13—C12—H12	126.2
C6—C5—C4	119.2 (3)	C11—C12—H12	126.2
C6—C5—H5	120.4	C12—C13—O3	110.1 (7)
C4—C5—H5	120.4	C12—C13—H13	124.9
C5—C6—C1	122.9 (4)	O3—C13—H13	124.9
C5—C6—H6	118.6		

Symmetry codes: (i) −*x*, *y*, −*z*+1/2.

### Hydrogen-bond geometry (Å, °)

**Table:**

*D*—H···*A*	*D*—H	H···*A*	*D*···*A*	*D*—H···*A*
C9—H9A···O1^ii^	0.97	2.32	3.292 (6)	177

Symmetry codes: (ii) −*x*, *y*−1, −*z*+1/2.

## References

[bb1] Aoki, S. & Kimura, E. (2004). *Chem. Rev.* **104**, 769–788. 10.1021/cr020617u 14871140

[bb2] Bruker (2000). *SMART*, *SAINT* and *SADABS* Bruker AXS Inc., Madison, Wisconsin, USA.

[bb3] Cai, Y., Wang, W., Qin, X., Li, Y. & Chen, W. (2010). *Z. Kristallogr. New Cryst. Struct.* **225**, 365–366.

[bb4] Camejo, J. J., Marrero, R., Gonzalez, C., Dominguez, J. A. & Castro, I. S. D. (1992). *Cana. Azucar.* **26**, 47–52.

[bb5] Csaszar, J., Morvay, J. & Herczeg, O. (1985). *Acta Phys. Chem.* **31**, 717–722.

[bb6] Greener, B., Moore, M. H. & Walton, P. H. (1996). *J. Chem. Soc. Chem. Commun.* pp. 27–28.

[bb7] Gultneh, Y., Ahvazi, B., Blaise, D., Butcher, R. J. & Jasinski, J. (1996). *Inorg. Chim. Acta*, **241**, 31–38.

[bb8] Ledovskikh, V. M. & Camejo, J. J. (1993). *Zashch. Met.* **29**, 597–603.

[bb9] Sheldrick, G. M. (2008). *Acta Cryst.* A**64**, 112–122. 10.1107/S0108767307043930 18156677

